# Results after spica cast immobilization following hip reconstruction in 95 cases: is there a need for alternative techniques?

**DOI:** 10.1007/s00402-020-03733-8

**Published:** 2021-01-11

**Authors:** L. Pisecky, G. Großbötzl, M. Gahleitner, C. Haas, T. Gotterbarm, M. C. Klotz

**Affiliations:** Department for Orthopaedics and Traumatology, Kepler University Hospital GmbH, Johannes Kepler University Linz, Kepler University Hospital GmbH, Krankenhausstrasse 9, Altenberger Strasse 69, 4040 Linz, Austria

**Keywords:** Spica cast immobilization, Complications, Hip reconstruction, NDH, DDH, Perthes disease

## Abstract

**Introduction:**

Developmental dysplasia of the hip (DDH), neurogenic dysplasia of the hip (NDH), and Perthes disease often require surgical treatment. Spica casting is a common postoperative immobilization.

The purpose of this study was to evaluate the complications related to the immobilization.

**Materials and methods:**

In a retrospective analysis, we included 83 patients (95 hips), who underwent hip reconstructive surgery between 2008 and 2018. We had 43 female and 40 male patients. Age reached from 3 months to 19 years. All patients were treated with a spica cast postoperatively for a 6-week protocol. Complications were analyzed using the full medical documentation and classified according to Clavien–Dindo.

**Results:**

We had complications in 23 patients (27.7%). We counted superficial skin lesions in seven, deep skin lesions in three, spasticity of adductors in three, subluxation in two, infection of the plate in one, fracture of the plate in one, compliance problem in one, dislocations of the cast in two, reluxation in one, delayed bone healing in one and spasticity of knee flexors in one case. According to the classification of Clavien–Dindo, we were able to count ten type I, four type II, nine type III, zero type IV and zero type V adverse events.

**Conclusion:**

The usage of a spica cast after hip reconstructive surgery is still the most popular way of aftertreatment. It has a low complication rate, which may be lowered by well-applied casts and foam padding. Known complications such as spasticity in patients with cerebral palsy, skin lesions, and pressure sores should be observed and avoided. Shorter protocols for immobilization with the usage of foam padding and foam splints lead to less complications.

**Clinical relevance:**

Evidence level level IV, case series

## Introduction

Developmental dysplasia of the hip (DDH) and neurogenic dislocation of the hip (NDH) require treatment to avoid impairment in walking, standing, and sitting [1]. Surgical hip joint reconstruction is the method of choice for children and adolescents with developmental dysplasia of the hip, as soon as conservative treatment has failed within the first postpartal months.

Severe forms of dysplasia of the pelvic joint are often related to neuromuscular disorders. While nonoperative treatment using braces, orthoses and physiotherapy are first-line therapy, some cases require surgical reconstruction of the hip. Especially children with neuromuscular disorders show a high incidence of NDH [2]. In cerebral palsy (CP), different authors could show an incidence of NDH in 18–60% of their patients [3].

In children with neuromuscular disorders, preventive surgery is recommended to avoid dislocation of the hip [4–6].

In most cases, a combination of procedures involving the bone and the soft tissue is needed to achieve open reduction of the joint [7, 8]. Common techniques are capsulotomy, lengthening or sectioning of shortened tendons or muscles, femoral and pelvic osteotomies. Aftertreatment is usually done by spica casting for 6 weeks, followed by physiotherapy [9].

Surgical treatment of Perthes disease aims to improve the containment of the pelvic joint. Indications for surgical treatment are the ‘head-at-risk signs’ with progressive loss of congruence of femur and acetabulum. Procedures used are pelvic and femoral osteotomies.

Most surgeons prefer casting to protect the osteosynthesis, the soft tissue and to avoid secondary dislocation due to spastictity. Possible complications are well known: hygienic problems, skin lesions, neurological complications, and rigidity of the joints after casting [10]. In common, a change of the spica cast is performed in a second short anaesthesia 2 weeks after surgery according to our aftertreatment regime. Due to the possible adverse effects of casting, alternative techniques of postoperative immobilization are discussed.

Retrospective studies showed the safety of the foam splint concerning the healing of the bone and promised less complications [10]. Despite retrospective data, there is no consensus about the way of postoperative immobilization.

The purpose of this study was to evaluate the short-term outcome after hip reconstruction in patients with DDH, NDH and Perthes disease. A further intent was to analyze the complications related to postoperative immobilization using spica casts and to suggest an alternative technique for postoperative immobilization and aftercare.

## Materials and methods

In a retrospective study, clinical records of children (age 0–19 years) with DDH, NDH and Perthes disease were screened for the presence of hip reconstructive surgery (86 patients). Included were children who received hip reconstructive surgery (open reduction, femoral and/or pelvic osteotomy ± soft tissue procedures) at Kepler University Hospital between 2008 and 2018. Patients who did not receive a spica cast immobilization after surgery (three patients) were excluded from further analyses. Finally, 83 children (male 40; female 43; 95 hips) with a mean age of 7.95 ± 5.18 years were included. For the patients with cerebral palsy, we used the Gross Motorfunction Classification System (GMFCS). There were 0 type I, 1 type II, 3 type III, 2 IV and 17 type V. In most of the patients, indication for surgery was DDH and Perthes disease, as seen in Table 1.

**Table 1:** Indications for surgical reconstrucion of the pelvic joint (*n* = 95)

	DDH > 6Mo	DDH < 6Mo	NDH	Perthes	Secondary dysplasia	Coxa vara	Coxa valga
*N*	36	4	23	25	5	1	1
Age at surgery	4.83y (2.6–17; SD 2.74; 95% CI 3.9–5.85)	3.8 m (0.22–0.44)	11.88y (5.4–19; SD 4.7; 95% CI 9.75–13.78)	7.41y (5–10.7; SD 1.53; 95% CI 6.67–8.05)	10.88y (4.6–18.7; SD 6.07)	15.4y	18.5y
M:f	5:19	0:4	12:10	21:3	2:3	1:0	0:1
Re:li	10:8	3:0	14:7	10:13	2:3	0:1	0:1
Bilat	9	1	1	1	0	0	0
GMFCS							
I	n.a	n.a	0	n.a	n.a	n.a	n.a
II	n.a	n.a	1	n.a	n.a	n.a	n.a
III	n.a	n.a	3	n.a	n.a	n.a	n.a
IV	n.a	n.a	2	n.a	n.a	n.a	n.a
V	n.a	n.a	17	n.a	n.a	n.a	n.a
Main diagnosis in detail							
Spastic CP			19				
Complex congenital malformation syndrome					1		
Trisomy 21					1		
Trisomy 18			2				
Trisomy 9					1		
Posttraumatic subluxation					1		
Dwarfism						1	
Subluxation after septic arthritis					1		
Muscular hypotonus in dysmorphic sondrome			2				
Surgical procedure in detail							
Femoral osteotomy	27		21	25	5	1	1
Salter osteotomy	12		3	12	2		1
Chiari osteotomy	1		3		1		
Pemberton osteotomy	19		8		1		
Psoas tenotomy			6		1		
Adductor tenotomy			4		1		
Open reduction	25	4	12		1		
Hamstring lengthening			7				
Lengthening of extension mechanism			1				

Identification of the patients was performed by a systematic filtered search of the surgical protocols of an University Hospital in central Europe.

Postpartal observed DDH with luxation of the hip (graf type IV) and failed closed reduction (four patients) underwent open reduction within the first 6 months (2.7–5.3 months). The cast was applied in human position (100 degrees of flexion, 50 degrees of abduction in the pelvic joint) for three (one patient) to 6 weeks (three patients) followed by application of Pavlik’s harness for 6 weeks.

None of the complications seen was observed in this subgroup.

Included indications for surgery are Reimers migration index 40% or higher or 25–40% with progression, Tönnis classification II or higher or AI (Acetabular index) above the Tönnis-standard. Surgery was not performed before the third year of age.

Surgical techniques used in NDH were derotating varisation osteotomy of the femur (21), Pemberton acetabuloplasty (8), Salter osteotomy of the acetabulum (3), and Chiari osteotomy (3). Soft tissue techniques were tenotomy of psoas muscle (6), of adductor muscle (4), of knee flexors, (7) and lengthening of quadriceps tendon (1). Mean age at surgery was 11.9 years (5.4–19; SD 4.7; 95% CI 9.75–13.78).

Surgical techniques used in DDH were derotating varisation osteotomy of the femur (27), Pemberton acetabuloplasty (19), Salter osteotomy of the acetabulum (12), and Chiari osteotomy (1). Mean age at surgery was 4.8 years (2.6–17; SD 2.7; 95% CI 3.9–5.85).

The surgeries were performed under general anesthesia on a radiolucent table using fluoroscopy. The patient was in supine position with mild elevation of side to operate on by placing a foam pad under the ilium. The entire lower limb and the affected half of the pelvis were washed and draped. The approach used for open reduction and surgical procedure of the ilium was an anterior approach (Smith–Petersen). The iliac apophysis was incised and withdrawn to expose the iliac bone. The approach to the proximal femur for varisation osteotomy was direct lateral.

Overall surgical techniques used were derotation varisation osteotomy in 79, Salter osteotomy in 30, Pemberton osteotomy in 28, open reduction in 42, Chiari osteotomy in 5 hips, valgisation osteotomy in 1 hip. In a total of 63 hips, a combination of the techniques above was performed.

Osteosynthetic material used to hold the femoral osteotomy was a conventional 90° AO blade plate (64 hips) or a 90° locking cannulated blade plate (15 hips).

The cast was applied in all cases directly postoperative in general anaesthesia. Staff involved in casting was: one senior surgeon, one junior surgeon, two theatre nurses, three casting professionals. Surgeons and nurses held the pelvis and lower extremities in the desired position, while the casting professionals applied two layers of cotton, followed by plaster. The reconstructed side was a long leg cast, the contralateral side a short leg. The cast was split on the operated side.

The position of the lower extremity operated on, was about 10 degrees of flexion as well as 10 degrees of inwards rotation of the hip and 20–30 degrees of abduction of the hip.

**Fig. 1:**
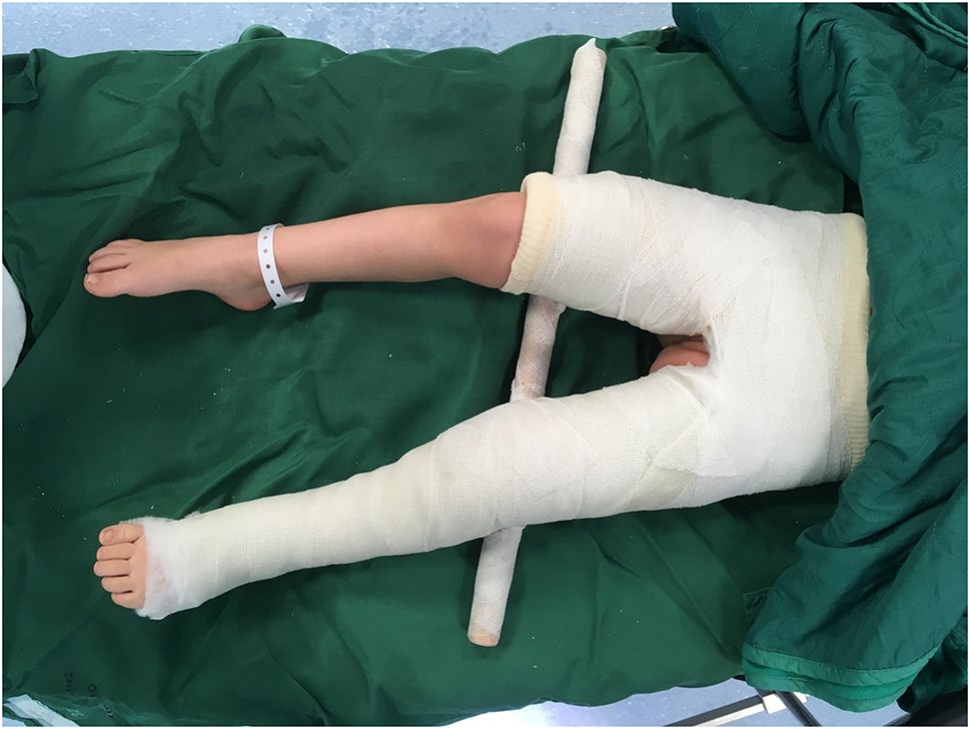
Applied spica cast with connecting rod

To keep abduction and for stabilization reasons, the thighs were connected by a rod. The final cast can be seen in Fig. 1.

According to our postoperative aftercare regime, sutures were removed and recasting was performed in a short general anaesthesia after 2 weeks.

All complications, which occurred within the first 3 months after surgery, were analyzed using the full medical documentation and classified according to Clavien–Dindo, as seen in Table 2 [11].

**Table 2:** Classification for surgical complications according to Clavien and Dindo (modified from Sink 2012) [24]

Grade	Definition
I	A complication that requires no treatment and has no clinical relevance; there is no deviation from routine follow-up during the postoperative period; allowed therapeutic regimens include: antiemetics, antipyretics, analgesics, diuretics, electrolytes, antibiotics, and physiotherapy
II	A deviation from the normal postoperative course (including unplanned clinic visits) that requires outpatient treatment: either pharmacologic or close monitoring as an outpatient
III	A complication that is treatable but requires surgical, endoscopic, or radiographic interventions or an unplanned hospital admission
IV	A complication that is life threatening, requires ICU admission, or is not treatable with potential for permanent disability; a complication that requires organ resection (THA)
V	Death

Absolute and relative frequencies of all seen complications were calculated, compared to existing literature and evaluated for plausibility.

The pre- and postoperative hip geometries were measured and compared statistically. The data obtained for hip geometry were: AI and CE (center-edge)-angle, Reimers migration index (RMI). X-rays used were the preoperative radiograph and the follow-up radiograph performed 3 months after reconstruction. Patients were categorized using the GMFCS scale.

### Statistical analysis

Statistical methods included a detailed descriptive epidemiological analysis with arithmetic mean, standard deviation, minimum, maximum, median at continuous data and scores, relative frequency for explained variables.

Tests for normal distribution (Shapiro–Wilk) were performed to show applicability of *t* tests. Normal distribution was shown for the selected parameters AI, CE, RMI. The variables for pre- and postoperative hip geometry are calculated using a paired samples *t* test (Wilcoxon rank). Subgroup analysis for AI, CE, and RMI was performed, using a paired samples *t* test (Wilcoxon rank). Values for *p* are given and values of < 0.05 are considered to be statistically significant. Whenever useful, graphics are used to illustrate the statistical results. The programs used for data analysis were Microsoft Excel version 16.27 and Jamovi version 1.0. Calculations were performed on MacOS Mojave Version Number 10.14.6.

## Results

Immobilization in spica cast was administered for a mean of 6.16 weeks, with a range from 5 to 12 weeks and a first standard deviation of 1.1 weeks.

In 23 patients, complications were seen (27.7%). We counted superficial skin lesions in 7, deep skin lesions in 3, spasticity of adductors in 3, subluxation in 2, infection of the implanted plate in 1, fracture of the plate in 1, compliance problem in 1, dislocations of the cast in 2, reluxation in 1, delayed bone healing in 1 and spasticity of knee flexors in 1 case, as seen in Table 3.

**Table 3:** Results and complications after surgery and immobilization at follow-up

	DDH > 6Mo	DDH < 6Mo	NDH	Perthes	Secondary dysplasia	Coxa vara	Coxa valga
N	36	4	23	25	5	1	1
Overall complications							
Superficial skin lesions	3		3	1			
Deep wound problems	1		1		1		
Spasticity of adductors			3				
Subluxation	1			1			
Reluxation	1						
Prolonged healing of bone					0		1
Plate fracture					1		
Plate infection	1						
Compliance problems				1			
Displaced cast	1			1			
Spasticity of knee flexors			1				
According to Clavien–Dindo							
I	4		3	3			
II			4				
III	4		1	1	2		1
Hip geometry							
Reimers preoperatively	80.28	n.a	78	27.38	57,33	n.a	30
Reimers postoperatively	2.77	n.a	12.94	1.75	17	n.a	10
AC-angle preoperatively	33.9	n.a	29.61	19	36.5	n.a	27
AC-angle postoperatively	19.43	n.a	18.56	16.71	29.67	n.a	16
CE-angle preoperatively	11.73	n.a	− 14.3	25.7	− 7.5	n.a	27
CE-angle postoperatively	31.7	n.a	27.5	32.9	23.67	n.a	42

According to the classification of Clavien–Dindo, we were able to count ten type I, four type II, nine type III, zero type IV and zero type V adverse events.

The breakdown of the complications by age can be seen in Table 4.

**Table 4:** Complications after surgery and immobilization at follow-up by age

Overall complications by age (years)	3–8	9–12	13–18
Superficial skin lesions	3	3	2
Deep wound problems	1	0	2
Spasticity of adductors	1	1	1
Subluxation	2	0	0
Reluxation	1	0	0
Prolonged healing of bone	1	0	0
Plate fracture	0	1	0
Plate infection	1	0	0
Compliance problems	1	0	0
Displaced cast	0	1	0
Spasticity of knee flexors	1	0	0
Clavien–Dindo I	4	4	2
Clavien–Dindo II	2	1	1
Clavien–Dindo III	5	1	3
Overall	11	6	6

The interventions needed to treat the complications were five surgical revisions with three repositions, two removals of the plate, three inpatient treatments for analgesia and wound care and one admission for high-energy shockwave treatment due to delayed healing of the bone.

Four patients needed wound treatment in our outpatient clinic on a continuous basis. In no case, very severe complications with persistent physical damage or even death occurred.

The study group considers nine type I, four type II und three type III complications to be associated with the used spica cast. Superficial (7), deep (3) ulceration, spasm of adductor muscles (3), spasm of knee flexor muscles (1), cast dislocation (1) and one case of difficulties with adherence to the cast immobilization were connected to the immobilization technique.

Surgical complications were: redislocation in three hips and infection of the plate in one hip.

In addition, two cases of complications, occurring in connection to the used osteosynthetic material were considered to be one case of delayed bone healing and one case of plate fracture, both 12 weeks after varisation osteotomy. All other cases showerd good bone healing after 12 weeks.

More often, complications were observed in the group of NDH (8/23; 34, 78%) than in DDH (8/36; 22, 2%), but the level of significance was not reached (*p* = 0.114).

Over all groups, the parameters for hip geometry improved statistically significant (*p* < 0.001), as seen in Table 3.

## Discussion

**Fig. 2:**
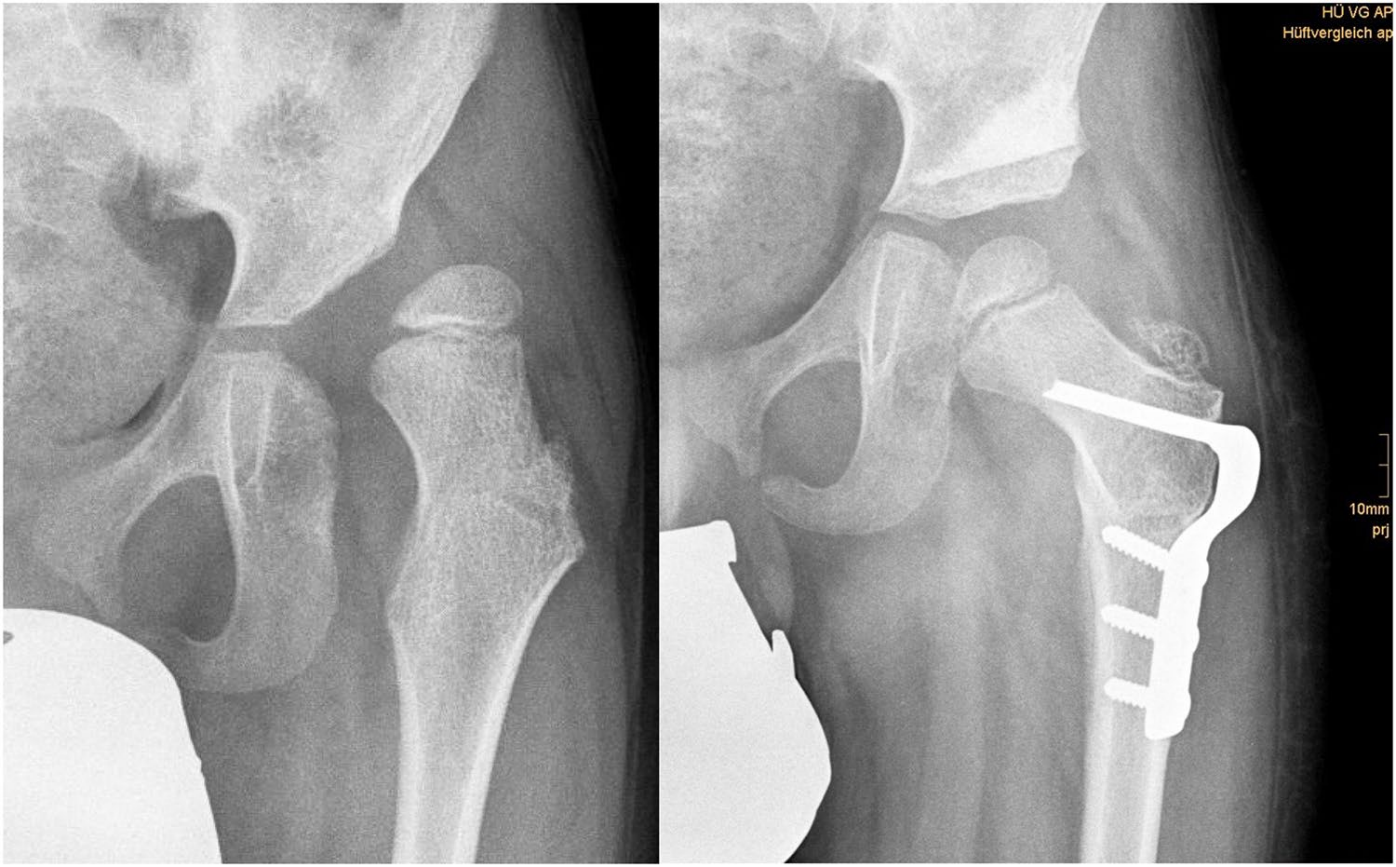
Anteriorposterior X-rays of a 6-year-old boy with neurogenic hip dislocation with excellent results 3 months postoperatively

Screening programs for DDH are well established in industrialised countries although the screening technique is not consistently clear [12]. It is commonly accepted that a surveillance program is mandatory to avoid progression of decentration and furthermore dislocation of the hip in children with NDH [13]. Therefore, it is clinical standard to evaluate hips at risk at least annually to prevent painful sub and dislocation. Failed conservative treatment necessarily leads to surgical procedures to reestablish congruency of the joint. Reconstruction of the pelvic joint under usage of pelvic osteotomies in combination with or without derotating varisation osteotomy and soft tissue techniques is a common way to treat DDH and NDH in children (Fig. 2).

The surgical results for reconstruction of the pelvic joint in this study group are comparable to previously published data by Czubak et al. 2018 [14], El-Sayed et al. 2015 [15],

Al-Ghamdi et al. 2012 [16] and Karlen et al. 2009 [17], who showed a preoperative AI of 37–39 degrees and an AI of 13–20 degrees postoperatively using periacetabular osteotomy.

The current study group states that the results for reconstruction of the pelvic joint using acetabuloplasty in the technique of Pemberton, pelvic osteotomy of Salter, Chiari osteotomy and varisation derotation osteotomy of the proximal femur are similar to the previously published data.

Indications for surgical procedures were developmental and neuromuscular dislocation of the hip as well as Perthes disease. For all patient groups, it was possible to show a statistically significant improvement in hip geometry achieved by hip reconstructive surgery as shown in Table 3.

Additional literature research revealed a complication rate of about 4.5 to 13.4 percent for spica cast after hip reconstructive surgery [18] and up to 28% in spica casts for femoral fractures [19].

Trying to find a simple way to avoid skin complications in patient undergoing pelvic reconstruction followed by cast immobilisation, Murgai et al. presented a retrosprective review of 920 patients with 2481 casts. Using foam padding, it was possible to reduce the complication rate in A-frame-casts from 13.4% to 4.5%, whereof the group of patients with encephalopathy had the lowest complication rate with foam padding (0.7%). Neurovascular deficits were described in none of the cases with foam padding compared to 4.5% in cases without foam [18].

The study group of DiFazio revealed a cast-associated complication rate of up to 28% in patients treated with spica casts for femur fractures, which lead to readmissions for recasting in 31% of those patients [19]. As a consequence of those findings, the same study group conducted a prospective clinical trial with foam padding and showed a reduction of complications from 13.6 to 6.6 cases per 1000 castings [20].

Previous studies, dealing with the optimal duration of aftercare following hip reconstructive surgery in children, showed, that a shorter period of immobilization using a spica cast leaded to less complications without risking the bone healing.

Up to now, the usage of a spica cast after hip reconstructive surgery, especially in spastic children, is still the most popular way of aftertreatment. Due to the well-known possible complications caused by a long-term casting and rigid immobilization, literature shows various attempts to establish alternatives in technique and duration of aftercare.

Shorter protocols for immobilization, as described by Emara 2018 [9], were investigated in clinical studies and published with positive results. In the discussed study protocol, the patients were included in a prospective clinical trial with a 4-week protocol, followed by abduction bracing, compared to a 12-week-protocol without further bracing. Results showed less complications and higher patient comfort in the group treated according to the shorter protocol. Besides, redislocation rates were not higher than in the group with longer immobilization.

A recent study of 2018 published by Alassaf tried to find out, whether a single-leg spica cast was able to provide enough stability for the immobilization period in children with late detected DDH and open reduction. In 162 hips (93 double, 69 single-leg), this retrospective review did not reveal statistical significance concerning complications caused by the type of immobilization and concluded, that there might not be a need for the casting of the contralateral side [21].

Gather, Dreher et al. made one step ahead and used a foam splint to establish an ultra-short immobilization protocol with early mobilisation and full weight bearing after 4 weeks [10]. In this clinical trial, including 33 hips, full weight bearing was allowed under control by a physical therapist after 4 weeks of immobilization in a foam splint. According to the authors of the study, no osseous complication or neurological complication caused by the foam splint was seen. This clinical trial leads to the conclusion, that foam splinting is at least as safe as spica casting concerning the postoperative osseous result.

Miller et al. presented their rather fast forward appearing postoperative regimen, where they do not use postoperative immobilization devices [22]. Immediate mobilization under physiotherapeutical company in 70 hips only lead to one revision due to persistent pain. Miller states to perform pelvic reconstruction without further immobilization and allows weight bearing after 2–4 days.

Two cases of complications occurring in connection to the used osteosynthetic material were considered to be one case of delayed bone healing and one case of plate fracture, both 12 weeks after varisation osteotomy. Rutz and Brunner showed healing rates of 100% in AO plates and LCP plates after 12 weeks in cases with cerebral palsy [23]. The introduction of a locking cannulated blade led to the replacement of the conventional AO blade plate, which was formerly used by the study group.

A wide range of possible aftercare regimes is given and in most cases, the personal preference of the surgeon and his experience is the reason why to choose one or the other. It is always a form of compromise to protect the osseo-capsular result of the sugery one one hand and to avoid skin problems or more severe complications due to long-term casting on the other hand. The surgeon’s intention to prevent the hip from redislocation stands in contrast to the patient’s and the caretaker’s wishes for a short, easy, and complication-free period of immobilization. Especially skin lesions, wound healing problems, and hygienic challenges are reasons for unplanned follow-up visits. Painful lesions of the skin up to deep ulcerations may need readmission to hospital and sometimes surgical revision. Unplanned readmissions and surgical procedured are severe interventions in the patient’s autonomy and the caretaker’s independence and shoul be avoided, whenever possible.

A divergence from the clinical pathway and readmission to hospital are classified as a type II and III complication according to Clavien–Dindo. Type IV and V complications with severe physical damage or even death were not seen in our cohort and not discussed in the available literature.

As type II and III complications lead to unexpected events, they should be avoided and so the search for alternative techniques and protocols in the aftercare of hip reconstructive surgery appears rewarding and necessary.

## Conclusions

Up to now, cast immobilization after hip reconstructive surgery is still a popular way of aftertreatment following hip reconstructive surgery.

Spica cast immobilization may lead to a variety of complications, which could be avoided by the usage of foam as a padding or as a foam splint device. The usage of alternative techniques for postoperative immobilization after hip reconstructive surgery has a benefit for the wound healing as well as for the prevention of neurological deficits, muscle spasms and shortening because of the accessibility of the joints for physical therapy. Data for the benefits of alternative techniques are already given and are promising for further improvements. Prospective randomized clinical trials on alternative techniques are required.
